# The relationship between serum 25-hydroxy vitamin D concentration and obesity in type 2 diabetic patients and healthy subjects

**DOI:** 10.1186/2251-6581-11-16

**Published:** 2012-09-21

**Authors:** Ehsaneh Taheri, Ahmad Saedisomeolia, Mahmoud Djalali, Mostafa Qorbani, Manouchehr Madani Civi

**Affiliations:** 1 Department of Nutrition and Biochemistry, School of Public Health, Tehran University of Medical Sciences, Tehran, Iran; 2 Department of Epidemiology & Biostatistics, School of Public Health, Tehran University of Medical Sciences, Tehran, Iran; 3 Endocrinology and Metabolism Research Center, Tehran University of Medical Sciences, Tehran, Iran; 4 National Iranian Oil Company(NIOC) Hospital, Tehran, Iran

**Keywords:** Vitamin D, PTH, Obesity

## Abstract

**Background:**

Both obesity and type 2 diabetes are associated with hypovitaminosis D. The aims of this study were to investigate the association of serum 25-hydroxy vitamin D (25(OH) D) and parathyroid hormone (PTH) concentration with body mass index (BMI) in type 2 diabetic patients compared to control subjects and their predicting role in obesity.

**Methods:**

This cross-sectional study was conducted on 200 subjects (100 type 2 diabetics and 100 healthy controls). Concentration of 25(OH) D, calcium, phosphorous, parathyroid hormone (PTH), fasting blood glucose, HbA_1_c, serum insulin, homeostasis model assessment of insulin resistance (HOMA-IR) was determined in the fasting samples. Anthropometric measurements including body mass index (BMI) were also measured.

**Results:**

Eighty-five percent of type 2 diabetics and 79% of healthy subjects were suffering from vitamin D deficiency or insufficiency. Serum concentration of 25(OH) D (22.08 ± 15.20 ng/ml) (r = −0.11**,** P = 0.04) and calcium (8.94 ± 0.59 mg/dl) (r = −2.25**,** P = 0.04) has significant statistically with BMI in type 2 diabetic patients. Serum concentration of PTH has non-significantly associated with BMI in diabetic patients and healthy subjects.

**Conclusion:**

Serum levels of vitamin D inversely and PTH positively are associated with BMI after adjusted for age, gender and serum calcium in both type 2 diabetic patients and healthy subjects. These associations were statistically significant for serum concentration of vitamin D and calcium only in diabetic patients. So the status of vitamin D is considered as an important factor in type 2 diabetic patients.

## Introduction

Nowadays we know obesity not only as a simple disease but also a most common and important risk factor of chronic disease such as diabetes, hyperlipidemia, cancers, cardiovascular disease and osteoarthritis [1]. Obesity is one of returnable environmental risk factor because it is created and treated with changes in life style. The incidence of obesity and overweight is rapidly increasing worldwide. World Health Organization (WHO) reported that the global prevalence of obesity will be increased from 350 million obese and 1 billion overweight [2] in to 2.16 billion overweight and 1.12 billion obese individuals in 2030. In Iran, there are 24.5 million obese and overweight people which are almost 30% of the population [3].

Vitamin D is a fat-soluble vitamin with hormonal functions. Vitamin D helps calcium and phosphorus homeostasis and bone metabolism [4]. Exposure to sunlight, dietary intake and supplementation with vitamin D is the main source of vitamin D in human [5]. Furthermore, vitamin D deficiency plays an important role in many other diseases such as diabetes [6], hypertension [7], cardiovascular disease [8], immune disorders, osteoporosis and cancers [9].

The Iranian Multi-Center Osteoporosis Study (IOMS) showed that 75.1% of women and 72.1% of men in Iran have vitamin D deficiency [10]. There are evidences that circulating levels of 25(OH) D has relationship with obesity. Some studies reported that serum concentration of 1, 25-dihydroxy vitamin D in obese people is higher than non-obese controls [11-13]. According to these studies, it is expected that obese subjects have an increased level of vitamin D. However, there are ample evidences demonstrating an inverse association between serum level of vitamin D and obesity [14-17]. Moreover, many studies suggested that either vitamin D deficiency or obesity is important risk factors of type 2 diabetes [18-20].

We know that homeostasis of calcium, vitamin D and PTH is related to each others. Therefore, we expected that there is association between obesity with serum concentration of calcium, vitamin D and PTH. The objective of our study was to determine the relationship between serum levels of vitamin D, PTH and BMI in type 2 diabetic patients compared to healthy controls.

## Methods

In this cross-sectional study, 180 subjects (aged between 20-80 years) including 100 type 2 diabetic patients from Iranian Diabetes association and National Iranian Oil Company (NIOC) - Central Hospital and 100 healthy subjects from staffs of Tehran University of Medical Sciences Tehran, Iran were selected via random sampling methods. Aged and gender was matched between two groups. Exclusion criteria were pregnancy, lactation, use of drugs which may affect lipid profile or calcium and bone metabolism, chronic disorders of liver and kidney, endocrinology disorders such as hypo- or hyper-thyroidism or parathyroidism, smoking, insulin injection, use of anti-convulsion drugs, use of vitamin D and calcium supplementation.

A written informed consent was taken from each participant after the full explanations about the study according to Tehran University of Medical Sciences Ethics Committee’s procedure.

After an overnight fasting, 10 ml of peripheral blood was taken. The blood samples were centrifuged at 3000 rpm for 10 min and stored at −20°C. Serum concentration of 25(OH) D was measured using chemiluminescence method. Serum level of PTH was measured using RIA kit (CIS Biointernational, France) with normal range of 8-79 pg/ml. Serum calcium and phosphorus were analyzed using Pars Azmoon kit (Pars Azmoon Co, Tehran, Iran). Normal range of calcium and phosphorous were defined to be 8.6 -10.3 mg/dl and 2.5 - 5 mg/dl, respectively.

Anthropometric data including weight and height were measured using Seca scale (Seca 725 GmbH & Co. Hamburg, Germany) while subjects wear light clothes and no shoes in standing posture. The accuracy of weight and height was 0.1 kg and 0.5 cm, respectively. Body mass index (BMI) was defined as weight (kg) divided to height squared (m^2^). Obesity was defined as BMI higher than 30 kg /m^2^. To decrease the seasonal variability in biochemical determinations, our sampling was performed between April to June 2010.

Statistical analyses were carried out using statistical package for social sciences (SPSS) Software, (SPSS Version 16, Chicago, USA). Normality of distributions was confirmed using histogram and Kolmogorov-Smirnov method before statistical analysis. All continuous variables are expressed as mean ± SD and categorical variables as number (%). The student’s t-test was employed to compare the differences between the mean of continuous variables. Linear regression with enter method was used to find association between BMI as dependent variable and age, serum concentration of calcium, PTH and vitamin D as explanatory covariates. The P- value less than 0.05 was considered as statistically significant.

## Results

Our study was performed on 180 individuals [52.7% of type 2 diabetic patients (52.6% men and 47.4% women) and 47.33% of healthy subjects (52.9% men and 47.1% women)].

Vitamin D deficiency defined with serum 25 (OH) D less that 50 nmol/l and serum 25 (OH) D between 50-75 nmol/l considered as vitamin D insufficiency.

The characteristics of study population including age, sex, anthropometrics measurements, serum levels of 25(OH) D, calcium, phosphorous and PTH and glycemic profile including fasting serum level of glucose, HbA_1_c, insulin and HOMA-IR are shown in Table 1. In our study the prevalence of vitamin D deficiency was 83.3% among diabetic patients and 75.6% among healthy subjects adjusted for age and sex. Standardized coefficients and correlation coefficients with BMI as a dependent variable in type 2 diabetic patients and controls are shown in Table 2. In diabetic patients and controls, serum levels of 25 (OH) D and calcium have inverse relationship with BMI adjusted for age, gender, serum calcium and serum PTH. These associations were significant in type 2 diabetes.

**Table 1:** Baseline demographic and biological characteristics of the overall population

**Variables**	**Type 2 diabetes**	**Control**	**P-value**
**Age (year)**	51.26 ± 11.18	51.55 ± 13.39	0.88
**Weight (kg)4**	76.96 ± 13.82	73.22 ± 12.98	0.09
**BMI (kg/m **^**2 **^**)**	26.22 ± 9.30	26.26 ± 4.55	0.98
**Calcium (mg/dl)**	8.94 ± 0.59	9.14 ± 0.53	0.02
**Phosphorous (mg/dl)**	3.66 ± 0.03	3.70 ± 0.04	0.59
**PTH (pmol/l)**	4.73 ± 18.80	45.98 ± 26.82	0.10
**25(OH) D (ng/ml)**	22.08 ± 15.20	22.22 ± 10.03	0.75
**FBS (mg/dl)**	64.18 ± 174.89	87.69 ± 9.58	<0.01
**HbA **_**1 **_**c (%)**	7.54 ± 1.93	4.97 ± 0.55	<0.01
**Insulin (μU/ml)**	11.78 ± 8.90	13.67 ± 18.15	0.13
**HOMA-IR**	89.45 ± 73.92	54.66 ± 31.04	<0.01

**Table 2:** **Pearson’s correlation coefficients *r*and standardized regression coefficients β between BMI as dependent variable and serum 25(OH)D, age, gender, serum calcium and serum PTH as independent variables in diabetic patients and controls**

	**Type 2 Diabetes**		**Controls**	
	**β**	***r***	**β**	***r***
**Age (years)**	0.06	0.03	0.08	0.03
**Gender (M/F)**	0.11	1.43	−0.16	−1.51
**Serum 25(OH)D (ng/ml)**	−0.02	−0.11*	−0.12	−0.04
**Serum calcium (mmol/l)**	−0.27	−2.81*	−0.26	−1.87
**Serum PTH (pmol/l)**	0.09	0.02	0.04	0.005

* P < 0.05.

Standardized β coefficients for PTH and explanatory variables are presented in Table 3. Serum levels of 25(OH) D and calcium in type 2 diabetic patients had statistically significant relationship with serum concentration of PTH.

**Table 3:** **Pearson’s correlation coefficients *r*and standardized regression coefficients β between PTH as dependent variable and BMI, age, gender, serum calcium and serum 25(OH) D as independent variables in diabetic patients versus controls**

	**Type 2 Diabetes**		**Controls**	
	**β**	***r***	**β**	***r***
**Age (years)**	−0.18	−0.33	−0.09	−0.30
**Gender (M/F)**	0.22	9.75	0.23	19.82
**Serum 25(OH)D (ng/ml)**	−0.30	−0.41*	−0.10	−0.30
**Serum calcium (mmol/l)**	−0.24	−2.25*	−0.25	−1.68
**BMI (Kg/m**^**2**^**)**	0.09	0.31	0.05	0.47

* P < 0.05.

## Discussion

This is the study in Iran determining the relationship between BMI and vitamin D levels. In our study, the prevalence of vitamin D deficiency was 82.1% in diabetic patients and 75.6% in healthy subjects. Iran is one of the sunny countries in the Middle East with high prevalence of vitamin D deficiency [10,21,22]. Our findings indicated that serum concentration of 25(OH) D correlated inversely with body mass index (BMI) in diabetic patients and healthy controls.

Our study indicated that there is a negative relationship between serum levels of 25(OH) D and BMI. There is a discrepancy in the literature about the association between obesity and serum level of vitamin D. Several studies, which published before 1990, indicated that serum concentrations of 1, 25-OH2-vit D in obese subjects were higher than non-obese controls. These studies had small sample size below 20 subjects [11-13,23]. The proposed explanation for increased body fat due to vitamin D incorporation is presented by Shi and colleagues. This study reported an increased 1,25OH2vit D intracellular calcium levels in cultured human adiposities due to vitamin D incorporation. Calcium stimulates lipogenesis and inhibits lipolysis which results in the accumulation of body fat [24,25].

However, contrary to these studies, many recent investigations have found a negative association between serum level of vitamin D and anthropometric measurements [16-19,24-26]. Furthermore, it has been proposed that obesity and fat accumulation decrease the bioavailability of vitamin D and trap 25(OH) D in fat tissue. Worstman, et al. after experimental B-irradiation demonstrated that concentration of vitamin D3 was 57% lower in obese than non-obese subjects, whilst there was no difference in the content of 7-dehydro cholesterol and it’s conversion to vitamin D3 among obese and non-obese subjects [26].

Blum, et al. with use of DXA and new liquid chromatography mass spectrometry (LC/MS) method measured vitamin D in serum and fat tissue in 17 obese men and women. Results of this investigation showed that fat tissue and serum level of vitamin D3 were inversely correlated both in obese and non-obese subjects [27].

In one study on 66 white Spanish women with BMI = 24-35 kg/m^2^, overweight and obese women are at higher risk of vitamin D deficiency, largely due to excess adiposity rather than inadequate intake [28]. Therefore body fat must be considered in relation between BMI and vitamin D. Another study on 250 over weight and obese adults of different ethnicities demonstrated that the serum level of vitamin D3 was inversely related to weight, higher waist circumference, and higher HbA_1_c, but not with adipose mass [29]. Pacifico and colleagues performed a study on 304 Caucasian children with overweight/ obesity and 148 healthy subjects with normal weight. It is disclosed that the serum level of 25(OH) D is inversely related to total adiposity, components of metabolic syndrome and hypertension [30]. Furthermore, there are several causes for different results in relation between obesity and vitamin D. Method and sensitivities of tests in measurement of serum 25(OH)D and 1,25(OH)_2_ D are different among studies. The older studies which show positive association, employed radio receptor assays for determination of serum level of vitamin D, whereas recent studies that show negative association between obesity and vitamin D used modern radioimmunoassay. However, season have no influence on the serum level of 1,25(OH)_2_D as it has on the serum 25(OH) D [31]. We selected the samples of this study in the wintertime in which insufficient vitamin D synthesis is very common. Accordingly, latitudes around 40° North or South have insufficient ultraviolet B (UV-B) radiation in winter time. Iran is located in 25° 39' 47'' latitude. Therefore, in sunny countries especially in Middle East such as Turkish [32], India [33], Kuwait [34] and Iran [22], vitamin D deficiency is very common due to the cultural and religious believes of women who cover their skin and have a limited sunlight exposure [35,36].

Regarding the relationship between vitamin D deficiency and obesity, there is a hypothesis called "winter response" which explains how vitamin D deficiency is causing obesity. This hypothesis explains that how difficult is to maintain body temperature and energy balance in a cold environment. Accumulation of fat tissue is causing to decreased heat conductance from body to environment [37], and blood circulation of skin [20]. Ultraviolet (UV) component of the sunlight acts as an environmental signal for season changes and skin synthesis of vitamin D which acts as a UV-radiation receptor. In this model, decreased concentration of 25 (OH) D causes to increase the set point of body weight in hypothalamus. Nowadays, inadequate dietary vitamin D and sunlight exposure, acts as a situation similar to the winter with winter response [38].

There are several proposed mechanisms about the inverse relationship between vitamin D and BMI. The studies were performed *in vitro* have been shown that vitamin D inhibits expression of uncoupling protein 2 (UCP2) in adipose tissue, differentiation of preadipocytes, synthesis and secretion of lipoprotein lipase [39]. UCPs are a family of inner mitochondrial membrane transporters having an important role in determination of resting energy expenditure (REE) [40]. Wong and colleagues by study on transgenic mice, showed that the prevalence of obesity was higher in mice with modified VDR (vitamin D receptor) compared to normal mice as a result of decreased REE and β-oxidation of fatty acids (41).

This study had some limitations. The main limitation of this study was a cross-sectional nature of this study, with no causality effect to report. The variation in polymorphism of DPB and VDR, sunlight exposure and effect of vitamin D supplementation on the weight gain are also need to be considered.

In conclusion, a serum level of 25(OH) D has inverse relationship with BMI among adult population with and without type 2 diabetes. This relationship was statistically significant in type 2 diabetic patients. However, it is not apparent either obesity is a direct consequence of vitamin D insufficiency or may result in vitamin D deficiency.

## Competing interests

The author(s) declare that they have no competing interests.

## Authors' contributions

MDJ, MMC contributed to the study design and supervised in biochemistry experiments; AS, ET carried out the experiments and provided the manuscript; MQ involved in the data analysis and the interpretation of results. All authors read and approved the final manuscript.
